# Supplementary material for: The association between walking speed and risk of cardiovascular disease in middle-aged and elderly people in Taiwan, a community-based, cross-sectional study
Source: PLoS One. 2020 Jul 6;15(7):e0235277. doi: 10.1371/journal.pone.0235277 (PMC7337282; doi:10.1371/journal.pone.0235277)
Supplement: Attachment — Submitted filename: PONE-D-20-04269--2020.3.10.doc [file pone.0235277.s001.doc]

The Authors propose the community-based study to explore the association between walking speed and cardiovascular disease (CVD) risk among community-dwelling middle-aged and elderly population in Taiwan. The study designs and methods used are basically appropriate, and the interpretations of the results are reasonable. However, there are several areas where the manuscript needs to be strengthened.

1.Please give the power of data collection.

2.Flow chart of selection of the study sample and procedure is suggested.

3.A statement including the reference number of the ethics committee where appropriate should appear in the manuscript.

4.From the epidemiologic viewpoint, there are many confounding factors in the evidenced-based researches. How the authors deal with associated confounding factors in this study?

5.The authors should point and clarify the feature and novel findings of this study.

6.Please consider the comparison with the other epidemiological studies in other areas using table so make clear the significance of this study.

7.The results of goodness-of-test in table 3 are suggested.

8.The authors should add the comments related to selection bias in this study to the perceived limitation subsection.

9.More discussion regarding the policy implications of their findings would be important for the use of methodology in health policy making.

10.Some references should be updated.

Totally, I would like to congratulate the authors for the enthusiasm invested in this study. However, the manuscript does not reach the level of quality required for publication as original article without major revision in PLOS ONE.
