# Supplementary material for: The association between walking speed and risk of cardiovascular disease in middle-aged and elderly people in Taiwan, a community-based, cross-sectional study
Source: PLoS One. 2020 Jul 6;15(7):e0235277. doi: 10.1371/journal.pone.0235277 (PMC7337282; doi:10.1371/journal.pone.0235277)
Supplement: Attachment — Submitted filename: response 3 edited dechart.docx [file pone.0235277.s002.docx]

**Responses to reviewers’ comments**

**Reviewer 1**

**Comment#1**

Please give the power of data collection.

**Response#:1**

We thank the reviewer for reminding us this important issue. The sample size determination was based on the G*power 3.1 software. We did calculate the minimum sample size before this study. We also added statements to explain the calculation in study design and participants. These statements read as: “Before this study, the minimum sample size for this study was calculated at the initial stage of the study after previewing a relatively smaller population. Considering an 85% power, a 95% confidence level, and 0.30 as the high CVD risk rate among middle-aged people, we calculated that 288 participants were required to detect differences between these two study groups with an odds ratio of at least 2.” (Lines 78-82).

1. Jacob C, *Statistical power analysis for the behavioral sciences*. International Biometric Society, 1988.

**Comment#2**

Flow chart of selection of the study sample and procedure is suggested

**Response#:2**

Thanks for the reviewer’s suggestion, we made the flow chart in response to this comment (Please see Appendix 1), and put it in manuscript as Figure 1.

**Comment#3**

A statement including the reference number of the ethics committee where appropriate should appear in the manuscript.

**Response#:3**

We thank the reviewer for reminding us this important issue. In response to this comment, the reference number is 102-2304B and we add it into our manuscript in study design and participants, which read as” The study was approved by the Chang-Gung Medical Foundation Institutional Review Board (102-2304B), and all the participants were fully informed and signed informed consent forms before enrollment.” (Lines 89-91)

**Comment#4**

From the epidemiologic viewpoint, there are many confounding factors in the evidenced-based researches. How the authors deal with associated confounding factors in this study?

**Response#:4**

Thanks for the reviewer’s viewpoint. We deal those confounding factor with logistic regression in Table 3. Sex, age, waist circumference, hypertension, diabetes mellitus and hyperlipidemia were used for adjustment. We add material in statistical analysis, which read as “Finally, the multivariate logistic regression model for predicting CVD risk and walking speed was adjusted for sex, age, WC, HTN, DM, and hyperlipidemia in each group.” (lines 121-122), and we add another material in result, which read as “The multivariate logistic regression model for predicting CVD risk and walking speed with an adjusted odds ratio (OR) was 0.127 (95% CI = 0.021-0.771) in middle-aged people with adjustment for sex, age, waist circumference (WC), hypertension (HTN), diabetes mellitus (DM), and hyperlipidemia (p < 0.05).” (lines 53-57)

**Comment#5**

The authors should point and clarify the feature and novel findings of this study.

**Response#:5**

We fully agree with the reviewer’s comment about novelty finding of our study, we have added statements in discussion, which read as: “Previous research has mainly focused on the relationship between walking speed and CVD risk [25] [26] [27] [28] in other countries. Our research was the first community-based study that examined the role of age in the relationship between walking speed and CVD risk in Taiwan. Thus, the novel findings of this study are the first to explore the association between CVD risk and walking speed in different age populations in Taiwan. Second, we conducted a community-based study and comprehensively collected various data from a health promotion project, which may have clinical implications.” (Lines 256-262).

**Comment#6**

Please consider the comparison with the other epidemiological studies in other areas using table so make clear the significance of this study.

**Response#:6**

We appreciate the reviewer’s comments and made a chart to compare with recent publication, and put it in manuscript as Table 4:

| title | author | date | Study design | finding |
| --- | --- | --- | --- | --- |
| Slow walking speed and cardiovascular death in well functioning older adults: prospective cohort study [25] | Julien Dumurgier.etc | 2009 | prospective cohort study | lower walking speed was strongly associated with cardiovascular mortality in a population of well functioning older people. |
| Association of walking speed in late midlife with mortality: results from the Whitehall II cohort study[26] | Alexis Elbaz.etc | 2013 | cohort study | slow walking speed during late midlife is associated with increased mortality over 6 years of follow-up |
| Objective measures of the frailty syndrome (hand grip strength and gait speed) and cardiovascular mortality: A systematic review [27] | Vinod Chainani.etc | 2016 | A systematic review | both gait speed and handgrip strength are associated with increased risk of cardiovascular mortality in diverse populations. |
| Treadmill walking speed and survival prediction in men with cardiovascular disease: a 10-year follow-up study [28] | Giorgio Chiaranda.etc | 2013 | Population-based prospective study | 1 km treadmill walking test at a moderate intensity is a useful tool for identifying mortality risk in patients with stable cardiovascular disease. |

**Comment#7**

The results of goodness-of-test in table 3 are suggested.

**Response#:7**

We appreciate the suggestion from reviewer. After a logistic regression model has been fitted, the p-value for Hosmer and Lemeshow goodness-of-fit test is less than 0.05, and we state it in Table 3 as “The p-value for Hosmer-Lemeshow goodness-of-fit test＞0.05” (Line 209)

**Comment#8**

The authors should add the comments related to selection bias in this study to the perceived limitation subsection.

**Response#:8**

We fully agree with the reviewer regarding the fact that our findings were obtained from community-based subjects and cannot be generalized to the whole middle-aged and elderly population in Taiwan. To response to the reviewer’s comment, this limitation has been acknowledged in the discussion section, which read as “However, our study still had limitations. The participants in our study were recruited from a relatively small group in northern Taiwan as our favored population. The characteristics of the residents from this small group might differ from characteristics in the general population, so the findings cannot be generalized to the whole middle-aged and elderly population in Taiwan. Selection bias should be considered, and the results of our study should not be extrapolated to other regions of Taiwan. Future studies using random sampling of communities with a wider range of regions would make the research more discursive.” (Lines 263-269).

**Comment#9**

More discussion regarding the policy implications of their findings would be important for the use of methodology in health policy making.

**Response#:9**

We thank the reviewer for this comment. We have added statements in the conclusion section for suggestions for further clinical practice of the results. These statements read as: “Thus, our findings may provide valuable information for clinicians to alert middle-aged people regarding the increased risk of CVD.” (Lines 275-276).

**Comment#10**

Some references should be updated.

**Response#:10**

In response to the reviewer’s suggestion, we have replaced some old references, those references are in number 11,18,21,22, and add extra reference for citation, those reference are in 25,16,27,28. Accordingly, we have changed the sequence of citations in the text.

**Reviewer 2**

**Comment:**

The authors aimed to evaluate the walking speed for predicting cardiovascular disease (CVD) risk in middle-aged and elderly people. I am interested in this topic, however, the data is mainly collected from a specific community. Why did the authors choose the residents living in Guishan district to be enrolled in their study? Can this small group represent and draw conclusion in whole middle-aged and elderly population of Taiwan including the downtown, suburban, and rural areas?

**Response#:**

We appreciate the reviewer’s concern. This is a community-based study, so we chose the participants living in Guishan district for convenient and selective sampling. The resident from this district might have different characteristics comparing with the general population, and we only recruit participants from this district in northern Taiwan as our favor population. Thus, the results can not represent the other regions of Taiwan. Future studies using random sampling of communities with wider range of area would make the research more robust. To response to the reviewer’s concern, this limitation has been acknowledged in the discussion section, which read as “However, our study still had limitations. The participants in our study were recruited from a relatively small group in northern Taiwan as our favored population. The characteristics of the residents from this small group might differ from characteristics in the general population, so the findings cannot be generalized to the whole middle-aged and elderly population in Taiwan. Selection bias should be considered, and the results of our study should not be extrapolated to other regions of Taiwan. Future studies using random sampling of communities with a wider range of regions would make the research more discursive.” (Lines 263-269).

**General Comments:**
I applaud the authors for their effort, however, the syntax, grammar and overall style of this manuscript is sometimes difficult to read and needs a robust editing from a fluent or native English speaker. For instance, in Introduction section, last sentence: “and elderly poeple.” is an obvious typo error. I suggest the authors to have the manuscript carefully proofread again to correct grammar and other errors.

**Response to General Comments:**

We thank the reviewer for reminding us this important issue. We had found a native English speaker to help us to edit our manuscript before we resubmitted it, and the English editing certificate was provided. (Please see Appendix 2)

**Major Comment 1:**
Please revise the title of your manuscript so that it contains details of the study design which characterize the investigation as well. Please provide more information on the novelty of your study. Introduction should finish with the aim of the study that should be described more clearly.

**Response to Major Comment 1:**
We appreciate the suggestion form reviewer. After we revised our manuscript, the title was rewrote as suggested in cover page. The title now reads as: “The Association between Walking Speed and Risk of Cardiovascular Disease in Middle-aged and Elderly people in Taiwan, a community-based, cross-sectional study”.

We fully agree with the reviewer’s comment about novelty finding of our study, we have added statements in discussion, which read as: “Previous research has mainly focused on the relationship between walking speed and CVD risk [25] [26] [27] [28] in other countries. Our research was the first community-based study that examined the role of age in the relationship between walking speed and CVD risk in Taiwan. Thus, the novel findings of this study are the first to explore the association between CVD risk and walking speed in different age populations in Taiwan. Second, we conducted a community-based study and comprehensively collected various data from a health promotion project, which may have clinical implications.” (Lines 256-262).

According reviewer’s helpful suggestion, we also modified our aim in the introduction, which read as “However, previous research has mainly focused on the relationship between walking speed and CVD risk only. Our research was the first community-based study that aimed to reveal the role of age in the relationship of walking speed and CVD risk.” (lines 72-75)

**Major Comment 2:**
There were total 400 participants in the project, which was a relatively small sample size. Sample size should be increased to claim scientific merits of your study. The claims made by authors are significantly weakened by the fact that they are talking about findings drawn from a small sample size, and then should have mentioned this as limitation without any justification. Did you calculate the sample size before the investigation and what should be the minimal sample size needed in this study to avoid underpowered? Could the authors show the power of their sample size?

**Response to Major Comment 2:**

We fully agree with the reviewer regarding the fact that our findings were obtained from community-based subjects and cannot be generalized to the whole middle-aged and elderly population in Taiwan. To response to the reviewer’s comment, this limitation has been acknowledged in the discussion section, which read as “However, our study still had limitations. The participants in our study were recruited from a relatively small group in northern Taiwan as our favored population. The characteristics of the residents from this small group might differ from characteristics in the general population, so the findings cannot be generalized to the whole middle-aged and elderly population in Taiwan. Selection bias should be considered, and the results of our study should not be extrapolated to other regions of Taiwan. Future studies using random sampling of communities with a wider range of regions would make the research more discursive.” (Lines 263-269). We also calculated sample size with G*power 3.1 software before this study. We did calculate the minimum sample size. We also added statements to explain the calculation in study design and participants. These statements read as: “Before this study, the minimum sample size for this study was calculated at the initial stage of the study after previewing a relatively smaller population. Considering an 85% power, a 95% confidence level, and 0.30 as the high CVD risk rate among middle-aged people, we calculated that 288 participants were required to detect differences between these two study groups with an odds ratio of at least 2.” (Lines 78-82).

1. Jacob C, *Statistical power analysis for the behavioral sciences*. International Biometric Society, 1988.

**Major Comment 3:**
Please indicate the selection of study participants by adding a CONSORT diagram with flow chart.

**Response# Major Comment 3:**
Thanks for the reviewer’s suggestion, we made the flow chart in response to this comment (Please see Appendix 1) ,and put it in manuscript as Figure 1.

**Major Comment 4:**
The Introduction and Discussion sections need to be expanded with improvements. Try to cite more references to support your Result. Moreover, some of the references are outdated. Please update your reference list.

**Response# Major Comment 4:**

In response to the reviewer’s suggestion, we add material in introduction, which read as” However, previous research has mainly focused on the relationship between walking speed and CVD risk only. Our research was the first community-based study that aimed to reveal the role of age in the relationship of walking speed and CVD risk.” (Line 72-75), we add material in discussion, which read as” Previous research has mainly focused on the relationship between walking speed and CVD risk [25] [26] [27] [28] in other countries. Our research was the first community-based study that examined the role of age in the relationship between walking speed and CVD risk in Taiwan. Thus, the novel findings of this study are the first to explore the association between CVD risk and walking speed in different age populations in Taiwan. Second, we conducted a community-based study and comprehensively collected various data from a health promotion project, which may have clinical implications. However, our study still had limitations. The participants in our study were recruited from a relatively small group in northern Taiwan as our favored population. The characteristics of the residents from this small group might differ from characteristics in the general population, so the findings cannot be generalized to the whole middle-aged and elderly population in Taiwan. Selection bias should be considered, and the results of our study should not be extrapolated to other regions of Taiwan. Future studies using random sampling of communities with a wider range of regions would make the research more discursive.” (Line 256-262, 263-269) We also have replaced some old references, those references are in number 11,18,21,22, and add extra reference for citation, those reference are in 25,16,27,28. Accordingly, we have changed the sequence of citations in the text.

**Appendix 1.**

Community screening in Guishan district, Taoyuan city.

Cluster-randomized sampling design (8 of 28 villages)

- Aged 50 years abd above
- Residents had lived in the community for half one year and above

Excluded:

- Unable to contact
- Disability
- Empty house

Available and ambulatory subjects (n=619)

Excluded (n=219)

- Decline to participate (n=196)
- Aged < 50 years old (n=12)
- Recent CVD (n=11)

Interviewed ambulatory subjects (n=400)

Excluded (n=91)

- Had a history of CVD (n=19)
- Aged 75 years and above (n=56)
- Unable to complete a 6-meter-long walk (n=14)
- Missing or incomplete data (n=2)

Subjects for final analysis (n=309)

**Fig 1. Flow chart of study subjects**

**Appendix 2.**
